# The effects of climate, catchment land use and local factors on the abundance and community structure of sediment ammonia-oxidizing microorganisms in Yangtze lakes

**DOI:** 10.1186/s13568-017-0479-x

**Published:** 2017-09-13

**Authors:** Xiaoliang Jiang, Yujing Wu, Guihua Liu, Wenzhi Liu, Bei Lu

**Affiliations:** 1 Key Laboratory of Aquatic Botany and Watershed Ecology, Wuhan Botanical Garden, Chinese Academy of Sciences, Lumo Road No. 1, Wuchang District, Wuhan, 430074 People’s Republic of China; 2 College of Life Sciences, University of Chinese Academy of Sciences, Beijing, 100049 China

**Keywords:** AOA, AOB, Land use, Nitrification, Submerged vegetation

## Abstract

Ammonia-oxidizing archaea (AOA) and bacteria (AOB) play important roles in regulating the nitrification process in lake ecosystems. However, the relative effects of climate, catchment land use and local conditions on the sediment ammonia-oxidizing communities in lakes remain unclear. In this study, the diversity and abundance of AOA and AOB communities were investigated in ten Yangtze lakes by polymerase chain reaction (PCR), clone library and quantitative PCR techniques. The results showed that the abundances of both AOA and AOB in bare sediments were considerably but not significantly higher than those in vegetated sediments. Interestingly, AOB communities were more sensitive to changes in local environmental factors and vegetation characteristics than were AOA communities. Amongst climate and land use variables, mean annual precipitation, percentage of agriculture and percentage of vegetation were the key determinants of AOB abundance and diversity. Additionally, total organic carbon and chlorophyll-*a* concentrations in lake water were significantly related to AOB abundance and diversity. The results of the ordination analysis indicated that 81.2 and 84.3% of the cumulative variance for the species composition of AOA and AOB communities could be explained by the climate, land use and local factors. The climate and local environments played important roles in shaping AOA communities, whereas catchment agriculture and water chlorophyll-*a* concentration were key influencing factors of AOB communities. Our findings suggest that the composition and structure of sediment ammonia-oxidizing communities in Yangtze lakes are strongly influenced by different spatial scale factors.

**Electronic supplementary material:**

The online version of this article (doi:10.1186/s13568-017-0479-x) contains supplementary material, which is available to authorized users.

## Introduction

Nitrification plays an important role as a link between nitrogen (N) inputs from anthropogenic sources and N losses by denitrification and anaerobic ammonium oxidation (Mulder et al. 1995). The rate-limiting step of nitrification, the oxidation of ammonia to nitrite, is performed by ammonia-oxidizing archaea (AOA) and bacteria (AOB) (Juretschko et al. 1998; Francis et al. 2005). Although both AOA and AOB can be detected in a wide range of aquatic habitats, their abundance varies widely (He et al. 2007). In most sediments, the abundance of AOA is greater than that of AOB (Bernhard et al. 2010; Hou et al. 2013; Shen et al. 2014). AOA and AOB have different physiological and metabolic characteristics, including their adaption to pH (Nicol et al. 2008), trophic status (Wu et al. 2010; Prosser and Nicol 2012), and ammonium concentration (Verhamme et al. 2011), leading to different community structures and activities of ammonia-oxidizing microorganisms in sediments.

Much evidence has shown that the abundance and community structure of ammonia oxidizers in sediments have significant relationships with water quality and sediment properties, such as pH, oxygen concentration, sediment moisture and organic matter (Guo et al. 2009; Hai et al. 2009; Hu et al. 2010; Cao et al. 2011; Wessen et al. 2011). Submerged macrophytes can influence the microbial community structure and abundance both directly and indirectly (Zhao et al. 2014). Plant N uptake is higher during early growth stages, which may lead to different N-use strategies between AOA and AOB (Thion et al. 2016). Furthermore, submerged plants can significantly influence the microbial community structure in sediments by releasing organic carbon (C) and altering the oxygen status (Herrmann et al. 2009; Long et al. 2016).

Anthropogenic disturbances, such as the conversion of vegetation land uses to cropland and urban areas in watersheds, have significant effects on environmental conditions and submerged plants in lakes, which may in turn change the abundance and community structure of ammonia oxidizers in sediments (Liu et al. 2015). Agricultural and urban land uses affect the lake water quality and sediment properties mainly by generating point and non-point source pollutants (Harding et al. 1998). In addition to generating pollution, impervious urban and built-up areas frequently alter the natural hydrological processes that may also influence the lake water quality (Liu et al. 2012). Climate strongly impacts the community structure of plants and animals, but its effect on ammonia-oxidizing microbial communities remains unclear (Fierer et al. 2009; Bru et al. 2011).

Until now, the relative effects of multi-scale factors on abundance and community structure of sediment ammonia-oxidizing microorganisms in lakes are poorly understood. In this study, the diversity and abundance of AOA and AOB communities were investigated in 10 Yangtze lakes by polymerase chain reaction (PCR), clone library and quantitative PCR techniques. The climate, catchment land uses and local factors (water quality, sediment properties and vegetation characteristics) were also measured. The aims of the present study were (1) to explore which factors are significantly correlated with the diversity and abundance of AOA and AOB in lake sediments and (2) to identify the total and relative contributions of each factor to the species composition of sediment AOA and AOB communities.

## Materials and methods

### Study sites and field sampling

**Fig. 1:**
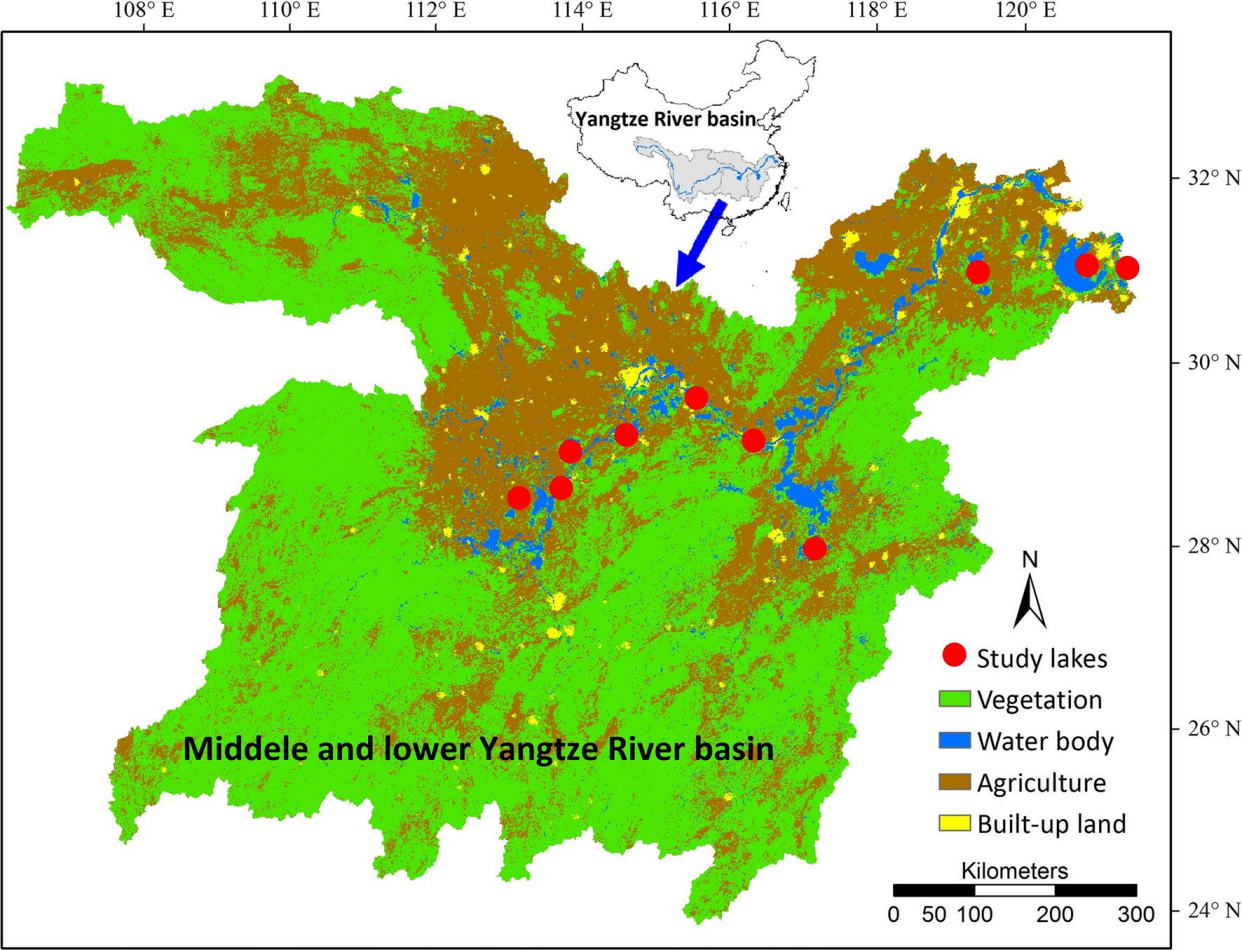
Location of ten study lakes in the Yangtze River basin

There are approximately 650 natural lakes (>1 km^2^ surface area) in the Yangtze River basin, China (Liu et al. 2015). Due to excessive nutrient inputs, eutrophication has become the primary water quality problem for most of the Yangtze lakes. In this study, we randomly selected ten shallow lakes in the middle and lower Yangtze River basin in July of 2013 (Fig. 1). The geographic location and morphological features of each lake are presented in Table 1. In each lake, sediment, water and submerged plant samples were collected from 3 to 4 sampling sites. These sites were chosen randomly for sampling but were separated by a minimum distance of 5 km. According to the status of submerged vegetation, these sampling sites could be classified into vegetated sites (N = 20) and bare sites (N = 15).

**Table 1:** The geographic location, morphology, climate, and catchment land uses of ten study lakes in the Yangtze River basin, China

Lake names	Longitude	Latitude	Elevation	Lake area	Mean depth	MAT	MAP	Catchment agriculture	Catchment built-up land	Catchment vegetation	Catchment water bodies
	(°E)	(°N)	(m)	(km^2^)	(m)	(°C)	(mm)	(%)	(%)	(%)	(%)
1. Lake Bajiaohu	113.20	29.45	27	12.3	3.0	16.50	1360	40.12	8.11	28.33	23.44
2. Lake Chihu	115.73	29.77	16	80.4	2.8	16.57	1583	46.27	2.36	34.56	16.81
3. Lake Dianshanhu	120.96	31.10	3	63.7	2.5	16.19	1311	54.11	2.75	33.19	9.95
4. Lake Donghu	112.65	29.38	25	23.2	3.5	15.26	1195	48.99	17.34	17.07	16.60
5. Lake Guchenghu	118.91	31.30	8	24.5	1.6	14.98	1292	44.13	3.25	41.13	11.49
6. Lake Honghu	113.36	29.84	25	344.4	1.9	16.24	1359	54.87	1.13	17.35	26.65
7. Lake Huahu	115.05	30.30	18	10.3	1.8	16.41	1532	43.22	6.67	33.21	16.90
8. Lake Junshanhu	116.36	28.51	18	192.5	4.0	17.15	1724	38.92	2.93	35.67	22.48
9. Lake Taihu	120.34	31.20	3	2425	2.1	15.26	1175	42.33	13.05	33.05	11.57
10. Lake Xilianghu	114.10	29.97	22	72.1	1.9	16.31	1451	37.33	2.43	37.17	23.07

MAT and MAP indicate mean annual temperature and mean annual precipitation, respectively

At each sampling site, triplicate sediment samples were collected randomly within an area of approximately 20 m^2^ using a homemade grab sampler. Surface sediments from a site were combined and homogenized in a bucket to form a composite sample. Approximately 12 g of homogenized sediments were placed into a centrifuge tube and then frozen in liquid N_2_ for molecular analysis. The remaining sediments were stored in a portable refrigerator at 5 °C for transport back to the laboratory. Water samples (500 mL) for water quality analyses were collected at approximately 0.5 m above the lake bottom before sampling sediments.

### Determination of local factors, catchment land use and climate

Water column pH, dissolved oxygen (DO), nitrate (NO_3_^‒^), and ammonium (NH_4_^+^) were measured in situ using a YSI 6920 multi-parameter water quality probe (YSI Inc., Ohio, USA). The total organic carbon (TOC) concentration of the filtered water samples was analysed by an elemental analyser (Vario TOC cube, Elementar, Germany). Water total phosphorus (TP) was determined by an acid digest followed by the colorimetric assay with the molybdenum blue method. The chlorophyll-*a* (Chl-*a*) concentration was measured by filtering 0.1‒0.2 L of water through 0.45-μm glass microfiber filters, followed by extraction with 90% acetone and measurement with a spectrophotometer (Beckman Coulter, Inc., Fullerton, USA).

Sediment moisture was measured after drying the wet sediments at 80 °C for 2 days, while sediment density was determined by weighing a known volume (50 cm^3^) of wet sediments. The contents of sediment total carbon (STC) and total nitrogen (STN) were analysed by an elemental analyzer (Vario TOC cube, Elementar, Germany) using air-dried and sieved (100-mesh) sediment samples.

In the field, submerged plant communities in each sampling site were investigated using a plant grab (25 × 35 cm) in the same areas of sediment sampling. The species richness and fresh biomass of submerged vegetation in each sampling site were determined according to Liu et al. (2015). Submerged plants were dried in an oven for 24 h at 65 °C and then ground to a powder. The concentrations of plant total N (PTN) and plant total phosphorus (PTP) were determined with the Kjeldahl method and molybdate-blue colorimetric method, respectively.

The percentage of agriculture, built-up land, vegetation and water bodies in each lake catchment were calculated in software ArcGIS 10.0 based on 2010 Landsat TM and HJ-1-CCD images (Table 1). A detailed description of the method has been provided in earlier studies (Liu et al. 2015, 2016). The mean annual precipitation (MAP) and mean annual temperature (MAT) of each sampling site were extracted from a 1 km resolution climate dataset from the Chinese ecosystem research network (Liu et al. 2010).

### Measurements of sediment ammonia-oxidizing communities

#### DNA extraction

Genomic deoxyribonucleic acid (DNA) was isolated from approximately 0.2 g of replicate sediment subsamples using the PowerSoil DNA Isolation Kit (MoBio Laboratories, Inc., Carlsbad, USA). The quality of the extracted DNA was checked in 1% TAE-agarose gel stained with Goldview and imaged under UV light. The DNA yield was quantified using NanoDrop 2000 fluorospectrometer (Thermo Fisher Scientific, Waltham, USA).

### Gene amplification, cloning, and phylogenetic analysis

The primers Arch-amoAF/Arch-amoAR (Francis et al. 2005) and amoA-1F/amoA-2R (Rotthauwe et al. 1997) were used to amplify the AOA and AOB *amoA* (ammonia monooxygenase subunit A) genes, respectively. The sequences of primers and thermal cycling procedures are shown in Additional file 1: Table S1. Each reaction was performed in a 25 μL volume consisting of 1 μL of DNA template (10–100 ng/μL), 0.5 μL of each primer (10 mM), 0.2 μL of rTaq polymerase (5U/μL) (TaKaRa, DaLian, China), 0.5 μL of deoxynucleotide triphosphates (10 mM) and 2.5 μL of 10× buffer. The PCR products were inserted into the pMD18-T vector (TaKaRa, DaLian, China) after gel purifying and transformed into Trans-5α competent cells (Transgen Biotech, Beijing, China).

Approximately sixty positive clones were screened by PCR and sequenced with an ABI-3730XL (Applied BioSystems, CA, USA) by Sangon Biotech Co., Ltd. After removing sequences of poor-quality or insufficient length by Geneious Pro 8.0.2 software (Biomatters Ltd., Auckland, New Zealand), the remaining sequences were aligned using the MAFFT software (Katoh et al. 2002). Operational taxonomic units (OTUs) with a more than 95% similarity level were calculated using Mothur software by the furthest neighbour algorithm (Schloss et al. 2009). Neighbour-joining phylogenetic trees were constructed with sequences from the main OTUs (containing at least two clones) and the most similar sequences retrieved from GenBank by software MEGA version 6. The diversity indices (i.e., Chao 1, Shannon–Weiner and Simpson) were calculated for each sediment sample using the software Mothur version 1.23.0.

The archaeal and bacterial *amoA* sequences obtained in this study have been deposited in GenBank with the following Accession Numbers: KU204801–KU204822, KY244149–KY244154, and KY244243–KY244299 for archaeal *amoA* and KU168323–KU168339 and KY244155–244242 for bacterial *amoA*.

### Real-time quantitative PCR

The abundances (i.e., copy number) of the *arch*-*amoA* and *amoA* genes were determined in triplicate using Roche LightCycler480 software version 1.5 with the fluorescent dye SYBR green quantitative PCR method. Primer sets of Arch-amoAF/Arch-amoAR and amoA-1F/amoA-2R were applied for the Arch-*amoA* and *amoA* genes, respectively. The 25 μL quantitative PCR mixture contained 10 μL of SybrGreen qPCR Master Mix (2×), 1 μL of primers (10 μM) and 2 μL of DNA template. The primers and qPCR thermal profiles are listed in Additional file 1: Table S1. Plasmids containing archaeal and bacterial *amoA* gene fragments were isolated using the SK8191 SanPrep Kit (Sangon Biotech Co., Ltd., Shanghai, China). Standard curves were constructed with serial plasmid dilutions of a known amount of plasmid DNA involving the target genes.

### Statistical analyses

Prior to statistical analysis, the distribution of the data was tested for normality using the Shapiro–Wilk test. Where necessary, data were natural log or square root transformed to achieve normal distributions. The relationships between ammonia-oxidizing communities and environmental factors were examined using Pearson’s correlation analysis with the PASW 19.0 software (IBM SPSS Inc., Chicago, USA). Additionally, a T test was used to examine differences in the diversity and abundance of nitrifiers between vegetated and bare sites. Canonical correspondence analysis (CCA) and redundancy analysis (RDA) were applied to quantify the influences of environmental factors on the species composition of AOA and AOB, respectively. A detrended correspondence analysis (DCA) was used to select the appropriate ordination analysis method (CCA or RDA). The statistical significance of the environmental factors was tested using Monte Carlo permutation tests in the software CANOCO 4.5 (Microcomputer, NY, USA).

## Results

### Climate, land use and local factors

The MAP ranged from 1175 mm in Lake Taihu to 1724 mm in Lake Junshanhu, with an average of 1394 mm (Table 1). The MAT varied between 14.98 °C in Lake Guchenghu and 17.15 °C in Lake Junshanhu. At the catchment scale, the percentage of agriculture ranged from a minimum value of 37.33% to a maximum value of 54.87%, whereas the percentage of natural vegetation varied from 17.07 to 41.13% (Table 1).

The water TOC concentration varied between 3.67 and 10.93 mg L^−1^ with a mean value of 6.64 mg L^−1^. The mean concentrations of water NO_3_^−^ and NH_4_^+^ were 0.11 and 0.26 mg L^−1^, respectively. The Chl-*a* content in the lake water showed a great variation, ranging from 1.38 to 78.77 mg m^−3^. In the sediments, the STC ranged from a minimum value of 5.6 to a maximum value of 104.2 mg g^−1^, while STN varied from 0.98 to 6.29 mg g^−1^.

### Abundance and diversity of AOA and AOB communities

The archaeal *amoA* gene abundance varied between 0.02 × 10^4^ and 333.34 × 10^4^ gene copies g^−1^ sediment (Additional file 1: Table S2), while the abundance of the bacterial *amoA* gene ranged from 0.17 × 10^4^ to 10.37 × 10^4^ gene copies g^−1^ sediment (Additional file 1: Table S3). The highest bacterial *amoA* and *arch*-*amoA* gene abundances were both observed in Lake Bajiaohu. The rarefaction curves showed that ammonia-oxidizing communities were sufficiently sampled to allow us to estimate the species diversity of AOA and AOB (Additional file 1: Figures S1, S2). The Shannon index of *amoA* gene ranged between 0.41 and 2.66 (Additional file 1: Table S2), while that of the *arch*-*amoA* gene varied from 0 to 2.58 (Additional file 1: Table S3).

There was no significant difference in diversity indices of AOA and AOB between vegetated and bare sites (Table 2). Similarly, the abundances of both AOA and AOB in bare sites were considerably but not significantly higher than those in vegetated sites. We found that AOB communities were more sensitive to changes in local environmental factors and vegetation characteristics than the AOA communities (Table 3). Amongst the climate and land use variables, MAP, percentage of agriculture and percentage of vegetation were the key determinants of AOB diversity. In addition, TOC and chl-*a* concentrations in lake water were significantly related to AOB abundance and diversity (Table 3).

**Table 2:** Diversity and abundance of nitrifying archaea and bacteria in sediments from vegetated and bare sites

	Vegetated sites (N = 20)	Bare sites (N = 15)	All sites (N = 35)
Chao1 diversity			
AOA	10.47 ± 8.92^b^	8.77 ± 7.36^b^	9.65 ± 8.12^b^
AOB	19.89 ± 12.18^a^	19.76 ± 16.22^a^	19.84 ± 13.83^a^
Shannon diversity			
AOA	1.31 ± 0.73	1.08 ± 0.49	1.20 ± 0.63
AOB	1.42 ± 0.66	1.51 ± 0.75	1.46 ± 0.69
Simpson diversity			
AOA	0.40 ± 0.27	0.47 ± 0.23	0.44 ± 0.25
AOB	0.41 ± 0.22	0.38 ± 0.26	0.40 ± 0.23
Abundance (10^4^ copies g^−1^)			
AOA	1.60 ± 1.36^b^	29.19 ± 86.27^b^	13.43 ± 57.07^b^
AOB	0.84 ± 0.70^a^	2.06 ± 2.83^a^	1.36 ± 19.99^a^
Abundance ratio			
AOA/AOB	2.79 ± 3.68	5.14 ± 8.32	3.79 ± 6.12

There was no significant difference in diversity and abundance of AOA and AOB between vegetated and bare sites. Mean ± standard deviation followed by different letters in the same column indicate significant difference (P < 0.05) between AOA and AOB

**Table 3:** Correlation between AOA and AOB community structure with climate, land use and local factors

	Chao1 diversity		Shannon diversity		Simpson diversity		Abundance		Abundance ratio
	AOA	AOB	AOA	AOB	AOA	AOB	AOA	AOB	AOA/AOB
MAT	−0.02	−0.27	−0.05	−0.15	0.02	0.20	0.15	0.25	0.10
MAP	0.07	−0.37*	0.09	−0.40*	−0.11	0.42*	−0.05	−0.08	−0.09
Agriculture	−0.23	0.39*	−0.35	0.44**	0.38*	−0.49**	−0.18	0.31	−0.33
Built-up land	0.01	0.02	−0.02	0.03	0.01	−0.01	0.10	−0.25	0.24
Vegetation	0.22	−0.33*	0.36*	−0.37*	−0.36*	0.34^*^	−0.08	−0.21	−0.01
Water bodies	−0.06	0.04	−0.10	0.04	0.07	0.06	0.21	0.21	0.14
pH	0.28	0.06	0.15	0.21	−0.16	−0.19	0.18	0.34*	0.20
Depth	0.14	−0.08	0.26	−0.12	−0.28	0.13	−0.04	0.09	−0.13
DO	0.28	0.03	0.24	0.23	−0.28	−0.21	0.04	0.26	−0.03
TOC	−0.34	0.46**	−0.29	0.46**	0.28	−0.49**	0.14	0.37*	−0.04
NH_4_^+^	−0.16	−0.35	−0.16	−0.23	0.10	0.17	−0.21	−0.10	−0.17
NO_3_^−^	−0.07	0.11	0.17	0.06	−0.21	0.00	0.31	0.12	0.39*
TP	−0.31	−0.01	−0.27	0.11	0.24	−0.20	0.07	0.25	0.00
Chl-*a*	−0.08	0.28	−0.02	0.39*	0.03	−0.37*	0.43*	0.65**	0.35*
Moisture	0.03	−0.11	0.22	−0.18	−0.26	0.16	−0.19	−0.26	−0.11
Density	−0.05	−0.09	−0.07	−0.02	0.08	0.02	0.11	0.06	0.09
STC	−0.29	0.10	−0.29	0.05	0.28	−0.08	−0.01	0.22	−0.11
STN	−0.12	0.00	−0.14	−0.09	0.12	0.10	−0.07	−0.01	−0.11
C/N	−0.40*	0.07	−0.35	0.08	0.33	−0.13	0.01	0.32	−0.11
Richness	0.10	0.00	0.14	−0.03	−0.13	0.01	−0.21	−0.22	−0.12
Biomass	0.09	0.19	−0.02	0.14	0.06	−0.11	−0.17	−0.11	−0.13
PTN	−0.14	0.56*	−0.07	0.46*	0.10	−0.49*	−0.15	0.52*	−0.30
PTP	0.18	−0.22	0.35	−0.11	−0.41	0.05	0.16	−0.25	0.43

** P < 0.01; * P < 0.05

### Species composition of AOA and AOB communities

**Fig. 2:**
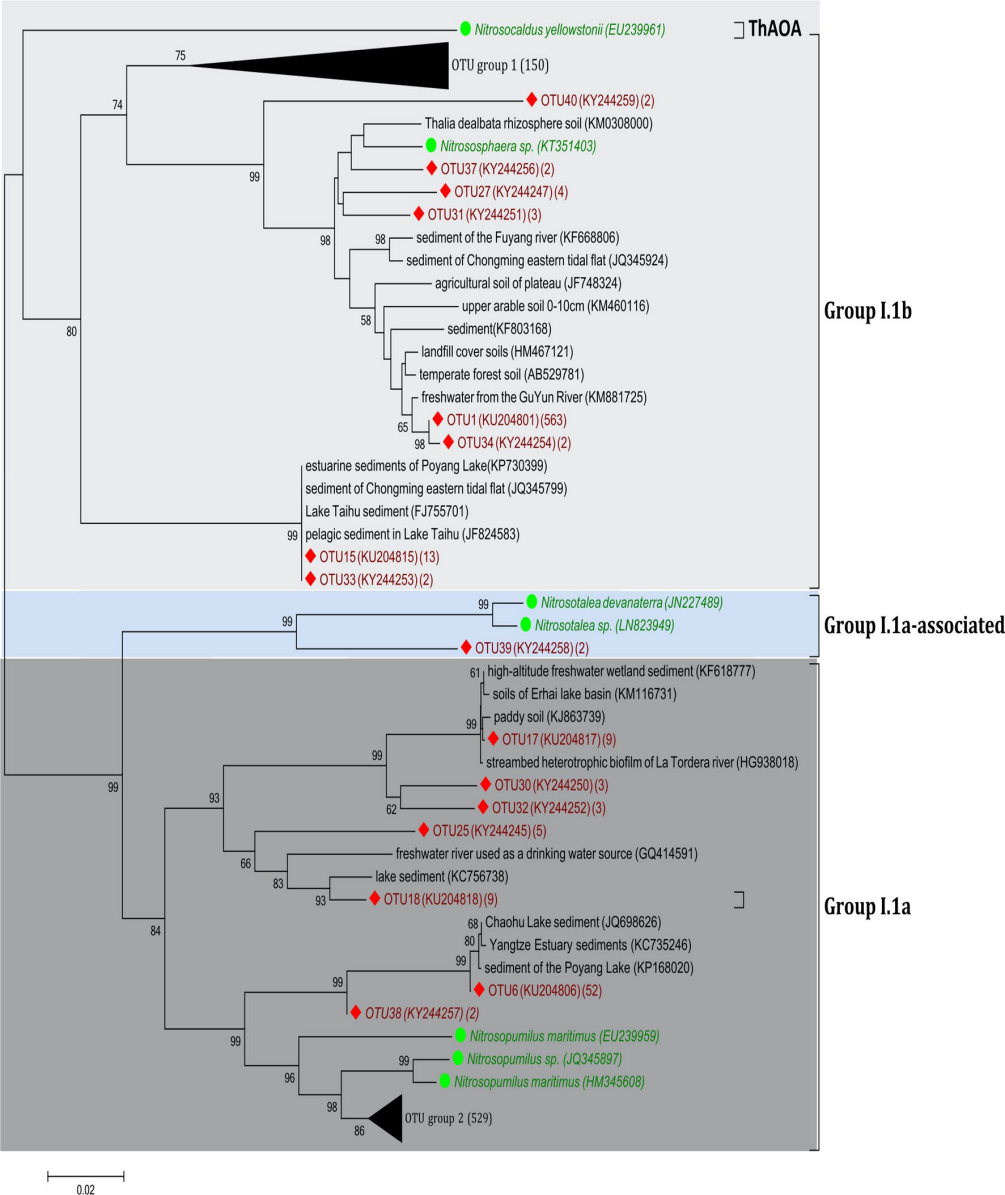
Neighbor-joining phylogenetic tree of archaeal *amoA*

**Fig. 3:**
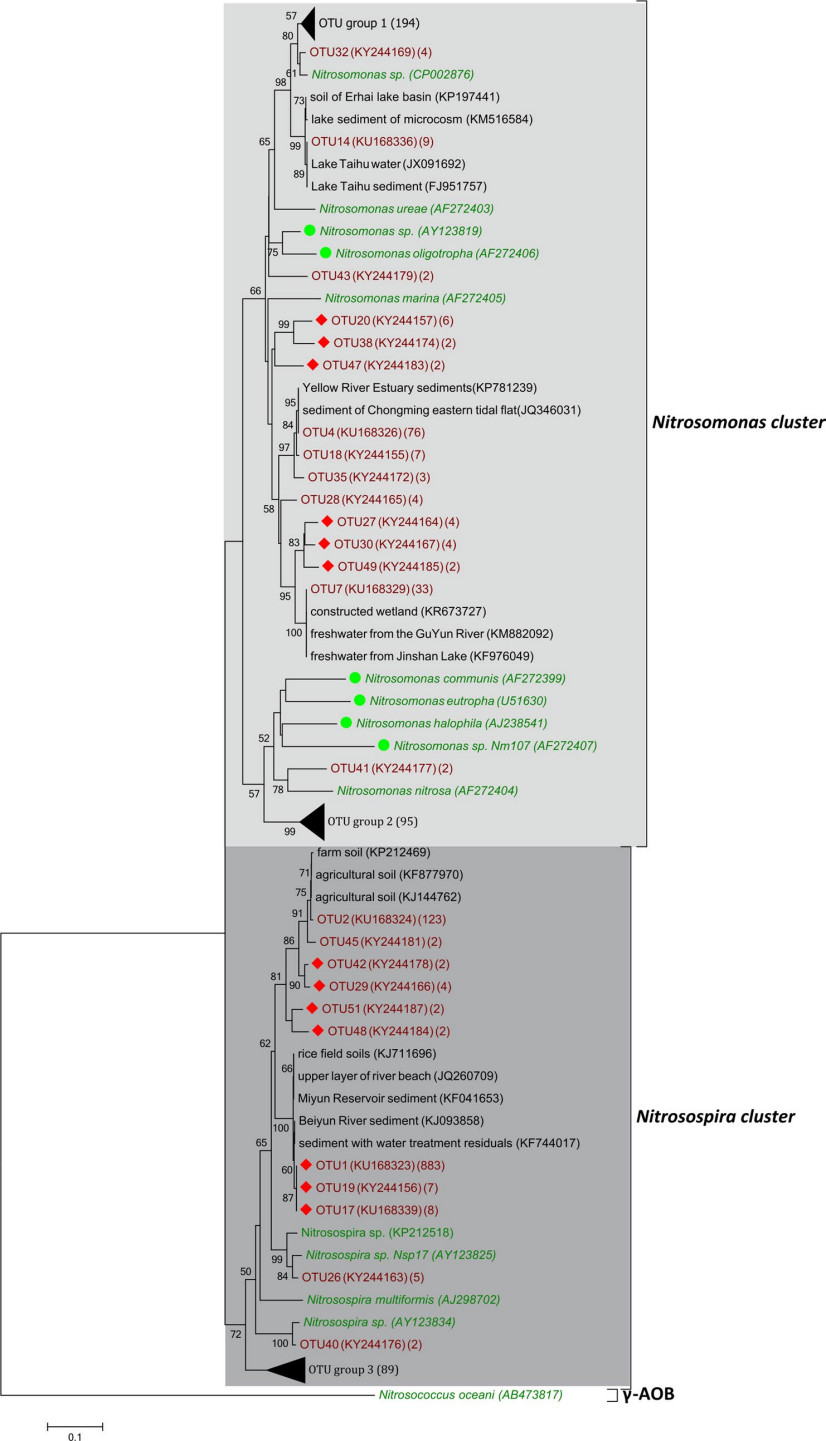
Neighbor-joining phylogenetic tree of bacterial *amoA*

A total of 1420 archaeal *amoA* clones and 1636 bacterial *amoA* clones were sequenced and grouped into 85 and 105 OTUs for AOA and AOB, respectively. The neighbour-joining phylogenetic tree showed that archaeal *amoA* gene sequences were grouped into three clusters (Fig. 2). Approximately 52.7 and 43.8% of the total archaeal *amoA* sequences fell into the group I.1b and group I.1a clusters, respectively. Only one OTU was affiliated with the group I.1a-associated cluster. For AOB, the *amoA* gene sequences resolved into three clusters, while approximately 69% of bacterial *amoA* sequences were affiliated with the *Nitrosospira* cluster (Fig. 3). The *Nitrosomonas* cluster was the second largest cluster, containing approximately 27.4% of the bacterial *amoA* sequences.

**Fig. 4:**
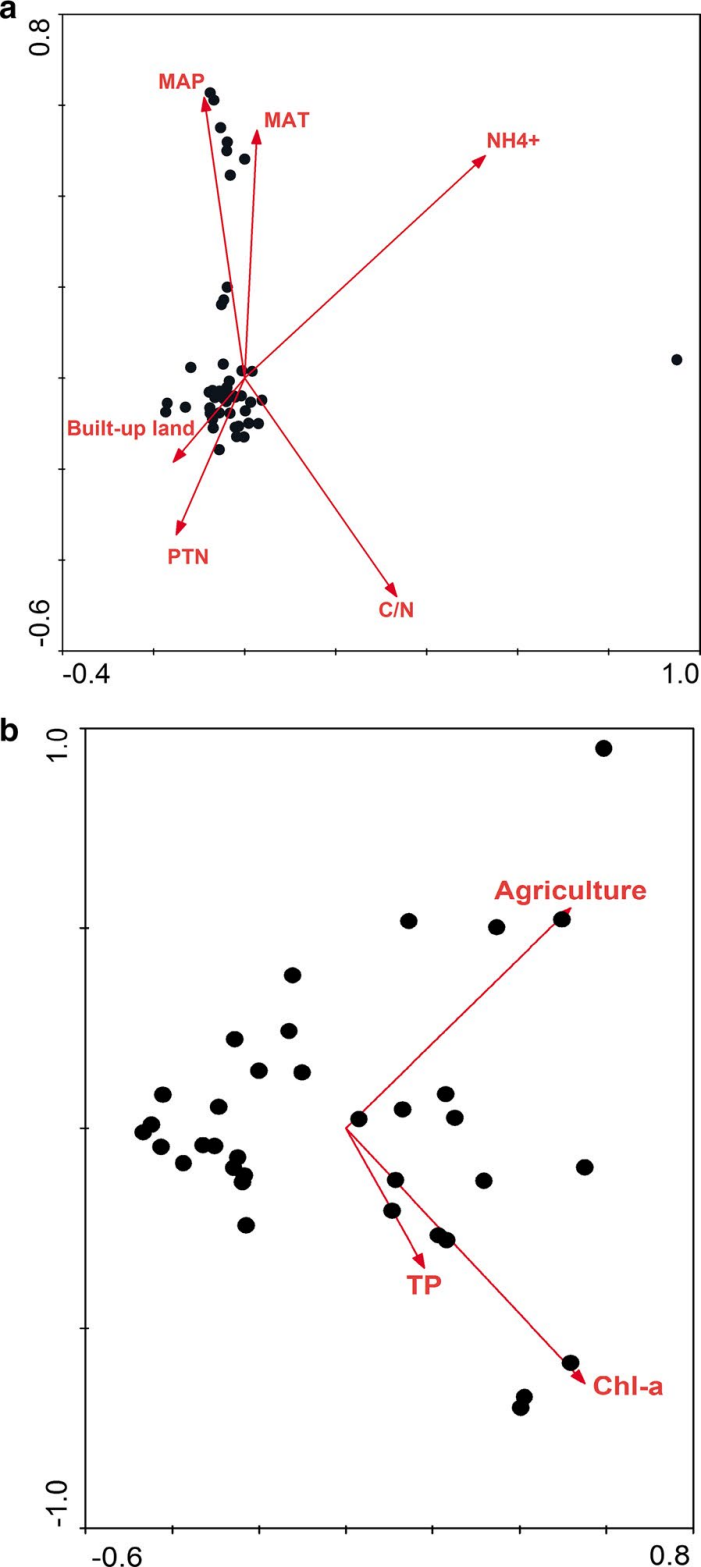
RDA or CCA ordination plots for the first two principal dimensions of the relationship between the AOB (**a**) and AOA (**b**) community compositions with the environmental parameters

CCA or RDA results showed that 81.21 and 84.30% of the cumulative variance for the species composition of AOA and AOB could be explained by the multi-scale factors (Fig. 4). Climate had a significant effect on the distribution pattern of the AOA community, with MAP and MAT significantly explaining 5.53 and 7.68% of the total variance, respectively (Table 4). Amongst local factors, water NH_4_^+^, sediment C/N and PTN could explain 7.06, 7.06 and 3.99% of the variation in the species composition of the AOA community, respectively. By contrast, the AOB community structure was mainly influenced by catchment land use and local water quality (Fig. 4b). The percentage of agriculture, water Chl-*a* and TP explained 22, 22 and 12% of the total variance in the AOB community (Table 4).

**Table 4:** Contributions of different factors to the variation in the community structure of AOA and AOB as revealed by canonical correspondence analysis or redundancy analyses

Variables	AOA		AOB	
	Variance explained (%)	P value	Variance explained (%)	P value
Climate				
MAT (°C)	*5.53*	*0.01*	2.00	0.30
MAP (mm)	*7.68*	*0.00*	1.00	0.36
Catchment land use				
Agriculture (%)	2.00	0.50	*22.00*	*0.00*
Built-up land (%)	*4.76*	*0.02*	2.00	0.23
Vegetation (%)	3.68	0.15	0.00	0.63
Water bodies (%)	0.00	0.55	3.00	0.09
Water quality				
pH	3.38	0.13	1.00	0.54
Depth (m)	3.38	0.13	2.00	0.11
DO (mg L^‒1^)	3.53	0.13	3.00	0.11
TOC (mg L^‒1^)	2.15	0.51	1.00	0.36
NH_4_^+^ (mg L^‒1^)	*7.06*	*0.00*	1.00	0.31
NO_3_^−^ (mg L^‒1^)	4.14	0.06	3.00	0.06
TP (mg L^‒1^)	3.22	0.24	*12.00*	*0.01*
Chl-*a* (mg m^−3^)	3.07	0.20	*22.00*	*0.00*
Sediment properties				
Moisture (%)	3.07	0.16	2.00	0.37
Density (g cm^−3^)	1.84	0.55	1.00	0.19
STC (mg g^−1^)	2.92	0.24	1.00	0.38
STN (mg g^−1^)	2.30	0.38	2.00	0.15
C/N	*7.06*	*0.00*	0.00	0.58
Vegetation characteristics				
Richness	2.46	0.40	1.00	0.44
Biomass (g m^‒2^)	1.23	0.78	1.00	0.35
PTN (mg g^−1^)	*3.99*	*0.04*	1.00	0.41
PTP (mg g^−1^)	2.76	0.24	0.00	0.63
All variables	81.21		84.30	

Significance was determined by 499 permutations

## Discussion

### Abundances of ammonia-oxidizing microorganisms

Previous studies indicated that AOA were numerically dominant over AOB in a variety of terrestrial and aquatic habitats, such as sheep farms (Li et al. 2016), eutrophic freshwater lakes (Hou et al. 2013; Zhao et al. 2014), arable lands (Jiang et al. 2014; Hu et al. 2015) and aquaculture ponds (Lu et al. 2015). Our study found that the average abundance of AOA was approximately 10 times higher than that of AOB (13.43 × 10^4^ versus 1.36 × 10^4^ copies g^−1^ sediment). Therefore, AOA might play an important role in regulating the ammonia oxidation processes of the Yangtze lakes. The ratio of AOA to AOB in sediments was influenced by several factors, such as pH, organic matter, and NH_4_^+^ (Dai et al. 2008; Wu et al. 2010; Zheng et al. 2013). In this study, the ratio of AOA to AOB was positively related to Chl-*a* and NO_3_^−^in the water column (Table 3), suggesting that the trophic status might be a key factor shaping the pattern of ammonia-oxidizing organism abundance (Hou et al. 2013; Bollmann et al. 2014; Yang et al. 2016).

The AOA and AOB abundances in soils or sediments were found to be significantly associated with pH (Sun et al. 2014), NH_4_^+^-N (Wu et al. 2007), NO_3_^−^-N (Liu et al. 2014), TN (Wang and Gu 2013), TP (Gan et al. 2016), and TOC (Verhamme et al. 2011). Consistent with Yao et al. (2011), we found that pH value was an important factor influencing the AOB but not AOA abundance in lake sediments (Table 3). This may be because AOA can distribute over a wide pH range with many populations adapted to highly acidic soils (Yao et al. 2011). In our study, the pH values of the sediment samples varied between 7.04 and 8.97, and the majority of samples exceeded 8. Previous studies have indicated that AOB dominate nitrification processes in alkaline or neutral environments whereas AOA may perform nitrification in acidic soils and sediments (Jiang et al. 2015). In this study, we also found that AOB abundance was positively related to TOC, Chl-*a* and PTN in Yangtze lakes (Table 3). This suggests that AOB are favored in environments characterized by high TOC and nutrients concentrations, which can lead to high mineralization and nitrification rates (Wessen et al. 2011).

Significant differences in AOA abundance were found between the algae-dominated and the macrophyte-dominated regions in eutrophic Lake Taihu (Dai et al. 2013). In addition, Zhang et al. (2015) reported that the AOA and AOB abundances in plant rhizosphere sediments were significantly higher than those in non-rhizosphere sediments. They explained that root exudates, such as plant hormones and carbohydrates, provided necessary nutrients for the growth of certain microbial communities in sediments (Garbeva et al. 2004). However, in our study, we found that the abundances of both AOA and AOB in bare sites were considerably but not significantly higher than those in vegetated sites (Table 2). In line with our results, Sun et al. (2014) reported that the abundances of AOA and AOB did not vary with ecological types (i.e., algae-type, macrophyte-type and transitional-type zones) in lake sediments. Our results may suggest that environmental factors, but not the presence of submerged vegetation, play the key roles in determining the abundance of ammonia-oxidizing microorganisms in Yangtze lakes.

### Effects of multi-scale factors on the diversity of AOA and AOB communities

It has been well documented that large-scale factors, such as climate and land use, play an important role in determining the diversity of macro-organisms such as animals and plants (Allen et al. 2002). However, the influence of climate factors on the abundance and diversity of sediment ammonia-oxidizing microorganisms has been little explored in the literature. In this study, we found that the Chao1 and Shannon diversity of AOB in lake sediments were negatively related to MAP (Table 3). Likewise, a recent study reported that MAT was significantly and negatively correlated with AOB but not AOA diversity in paddy soils (Hu et al. 2015). The different response of AOB and AOA to climate variables may be explained by their intrinsic physiological and genetic differences. AOB are highly sensitive to water stresses that may impact microorganism activity through substrate limitations and dehydration, whereas AOA had a strong tolerance to climate change (Chen et al. 2013).

Small changes in catchment land uses may lead to great changes in lake water quality and sediment physicochemical properties (Liu et al. 2015). Thus, catchment land uses are likely to indirectly influence the community structure of ammonia oxidizers in lake sediments through altering the water quality and sediment characteristics. In our study, we found that agriculture and vegetation land uses were significantly related to the diversity of AOB (Table 3). In the Yangtze River basin, agriculture is considered the main source of nonpoint-source pollution and has caused a number of environmental problems (Xiong et al. 2015). Agricultural land uses in catchments can increase C inputs to lakes as a result of increased soil erosion and organic matter transport or increased plant productivity that increases the rates of organic matter deposition to lake sediments (Liu et al. 2016).

Some studies reported that N-related variables were the key important factors determining the diversity of AOB in soils or sediments since NH_4_^+^ was the primary energy source for ammonia oxidizers (Verhamme et al. 2011; Liu et al. 2014). However, in this study, no significant correlation was observed between N-related variables and the diversity of ammonia-oxidizing microorganisms (Table 3). The detected relationship between TOC and AOB diversity was unexpected because most AOB were obligate chemolithotrophic bacteria (Zheng et al. 2013). However, Antje and Heinz (1982) found that the growth of many ammonia oxidizers was significantly enhanced by organic matters, although some species had low tolerance to organic compounds. Nevertheless, our results here provide evidence that AOB communities were more sensitive to changes in local environmental factors than AOA communities.

### Phylogenetic analysis and community composition of AOA and AOB

The phylogenetic tree indicated that the sediment AOA community was dominated by the group I.1b cluster (Fig. 2), a finding consistent with previous studies conducted in terrestrial and freshwater habitats (Jiang et al. 2014; Sun et al. 2014). In addition, approximately 43.8% of archaeal *amoA* sequences were affiliated with the group I.1a cluster (also called the “marine” group). In general, group I.1a existed mainly in oceans, except for *Nitrosopumilus* sp., which was abundant in agricultural soils (Jung et al. 2011). In the present study, we also found that the *Nitrosopumilus* group was present in sediments of freshwater lakes in the Yangtze River basin (Fig. 2). The phylogenetic tree indicated that the sediment AOB community in the Yangtze lakes was dominated by the *Nitrosospira* cluster (Fig. 3). Although both *Nitrosospira* and *Nitrosomonas* were ubiquitously present in freshwater sediments (Liu et al. 2014; Yao and Peng 2017), *Nitrosospira* was found to be more advantageous in various habitats such as agricultural soils (Wessen et al. 2010), peaty soils (Ciccolini et al. 2016), meadow soils (Avrahami and Conrad 2003) and the rhizosphere sediments (Zhang et al. 2015).

A number of environmental factors can impact the community composition of AOA and AOB, and their influences are complicated and mutable (Zheng et al. 2013). Recent investigations have reported that climate conditions were important in regulating the AOA and AOB community compositions (Angel et al. 2010; Jiang et al. 2014; Hu et al. 2015). Similarly, we found that MAT and MAP had a significant effect on the community structure of AOA but not AOB (Fig. 4). Consistent with Nielsen et al. (2010), we also found a significant relationship between archaeal community composition and sediment C/N and water NH_4_^+^. The C/N ratio is a direct measure of resource quality and can reflect the N availability in sediments. When the sediments have a high C/N ratio, N demand by ammonia-oxidizing microorganisms is high and AOA and AOB might compete for available NH_4_^+^ (Adair and Schwartz 2008). Moreover, AOA had a more competitive advantage than AOB in the uptake of NH_4_^+^ when the NH_4_^+^ concentration was relatively low (Yu et al. 2016).

RDA indicated that TP was a key determinant of AOB community composition, which was in agreement with the previous reports (e.g., Zheng et al. 2014; Yang et al. 2016). P is recognized as an essential nutrient element for almost all organisms, including AOB. Lage et al. (2010) found that changes in community composition of AOB under P amendment might reflect changes in interactions with other organisms (e.g., plants and heterotrophic fungi) that were P-limited. In addition, Zheng et al. (2014) reported that the relationship between TP and community compositions of ammonia oxidizers could be ascribed to the changes of nutrient ratios resulting from different levels of P that might impact the growth of ammonia oxidizers. Until now, little has been known about the influences of the water Chl-*a* concentration on the community composition of ammonia-oxidizing microorganisms. Consistent with a previous study conducted in the Derwent Estuary, Australia (Abell et al. 2013), we found that the Chl-*a* concentration had a significant relationship with the AOB community composition in Yangtze lakes. This result was unexpected, but may be explained by the fact that the overgrowth of algae will change the redox conditions, which may in turn affect the composition of sediment AOB communities.

In conclusion, by using PCR, clone library and quantitative PCR techniques, we investigated the abundance, diversity and community composition of ammonia-oxidizing microorganisms in 35 sediment samples from ten shallow lakes in the Yangtze River basin. We found that there were no significant differences in the abundance and diversity indices of AOA and AOB between bare and vegetated sediments. The average abundance of AOA was approximately 10 times higher than that of AOB, suggesting that AOA might play an important role in regulating the ammonia oxidation processes of the Yangtze lakes. We also found that the diversity indices of AOB but not AOA were strongly influenced by different spatial scale factors such as MAP and TOC. The ordination analysis indicated that 81.2 and 84.3% of the cumulative variance for the species composition of AOA and AOB communities could be explained by the climate, land use and local factors.

## Additional file

**Additional file 1. Figure S1:** Rarefaction curves of the amoA gene sequences of AOA obtained from 35 lake sediments. **Figure S2**: Rarefaction curves of the amoA gene sequences of AOB obtained from 35 lake sediments. **Table S1**: Primers and conditions for qualitative and quantitative PCR. **Table S2**: Abundance and diversity indices of clone libraries of archaeal amoA genes in different sampling sites. **Table S3**: Abundance and diversity indices of clone libraries of bacterial amoA genes in different sampling sites.

## Abbreviations

AOA: ammonia-oxidizing archaea
AOB: ammonia-oxidizing bacteria
PCR: polymerase chain reaction
N: nitrogen
C: carbon
DO: dissolved oxygen
NO_3_^‒^: nitrate
NH_4_^+^: ammonium
TOC: total organic carbon
TP: total phosphorus
Chl-*a*: chlorophyll-*a*
STC: sediment total carbon
STN: sediment total nitrogen
PTN: plant total nitrogen
PTP: plant total phosphorus
MAP: mean annual precipitation
MAT: mean annual temperature
DNA: deoxyribonucleic acid
*amoA*: ammonia monooxygenase subunit A
OTUs: operational taxonomic units
CCA: canonical correspondence analysis
RDA: redundancy analysis
DCA: detrended correspondence analysis
