# Supplementary material for: The effects of climate, catchment land use and local factors on the abundance and community structure of sediment ammonia-oxidizing microorganisms in Yangtze lakes
Source: AMB Express. 2017 Sep 13;7:173. doi: 10.1186/s13568-017-0479-x (PMC5597559; doi:10.1186/s13568-017-0479-x)
Supplement: Supplementary file 1 — Additional file 1. Figure S1: Rarefaction curves of the amoA gene sequences of AOA obtained from 35 lake sediments. Figure S2: Rarefaction curves of the amoA gene sequences of AOB obtained from 35 lake sediments. Table S1: Primers and conditions for qualitative and quantitative PCR. Table S2: Abundance and diversity indices of clone libraries of archaeal amoA genes in different sampling sites. Table S3: Abundance and diversity indices of clone libraries of bacterial amoA genes in different sampling sites. [file 13568_2017_479_MOESM1_ESM.doc]

**Additional File**

**Figure S1** Rarefaction curves of the *amoA* gene sequences of AOA obtained from 35 lake sediments.

**Figure S2** Rarefaction curves of the *amoA* gene sequences of AOB obtained from 35 lake sediments.

**Table S1** Primers and conditions for qualitative and quantitative PCR.

**Table S2** Abundance and diversity indices of clone libraries of archaeal *amoA* genes in different sampling sites.

**Table S3** Abundance and diversity indices of clone libraries of bacterial *amoA* genes in different sampling sites.


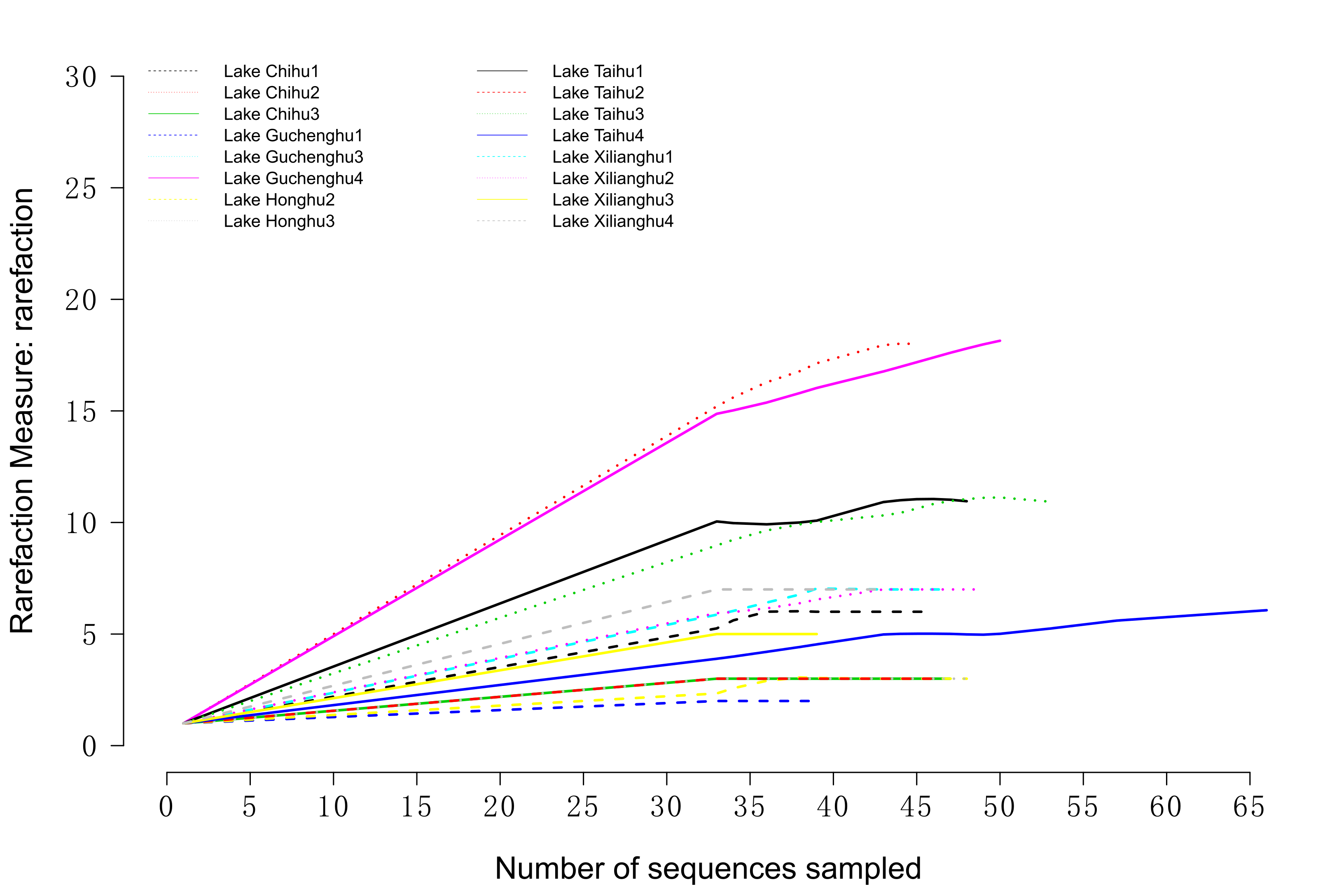

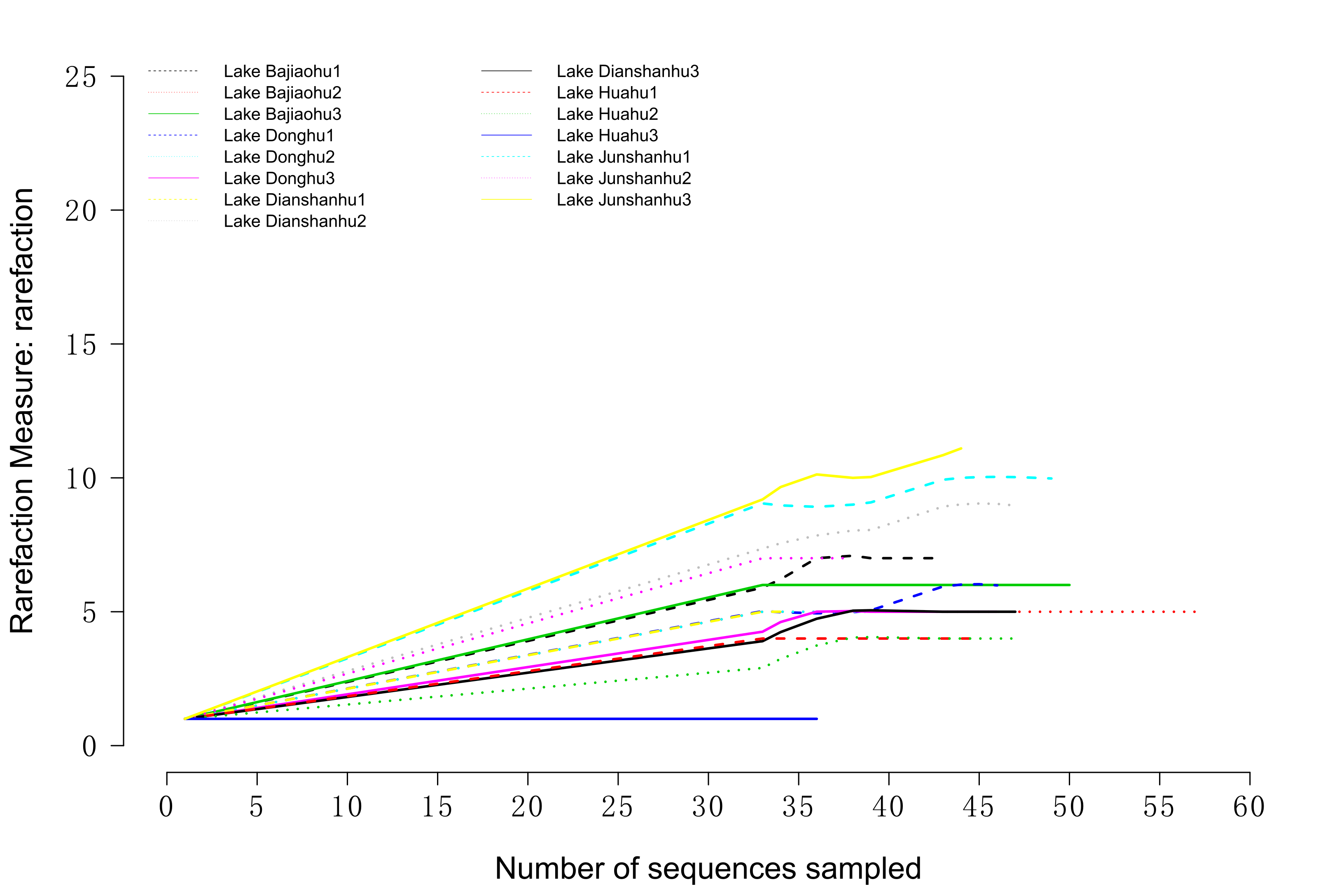


**Figure S1**


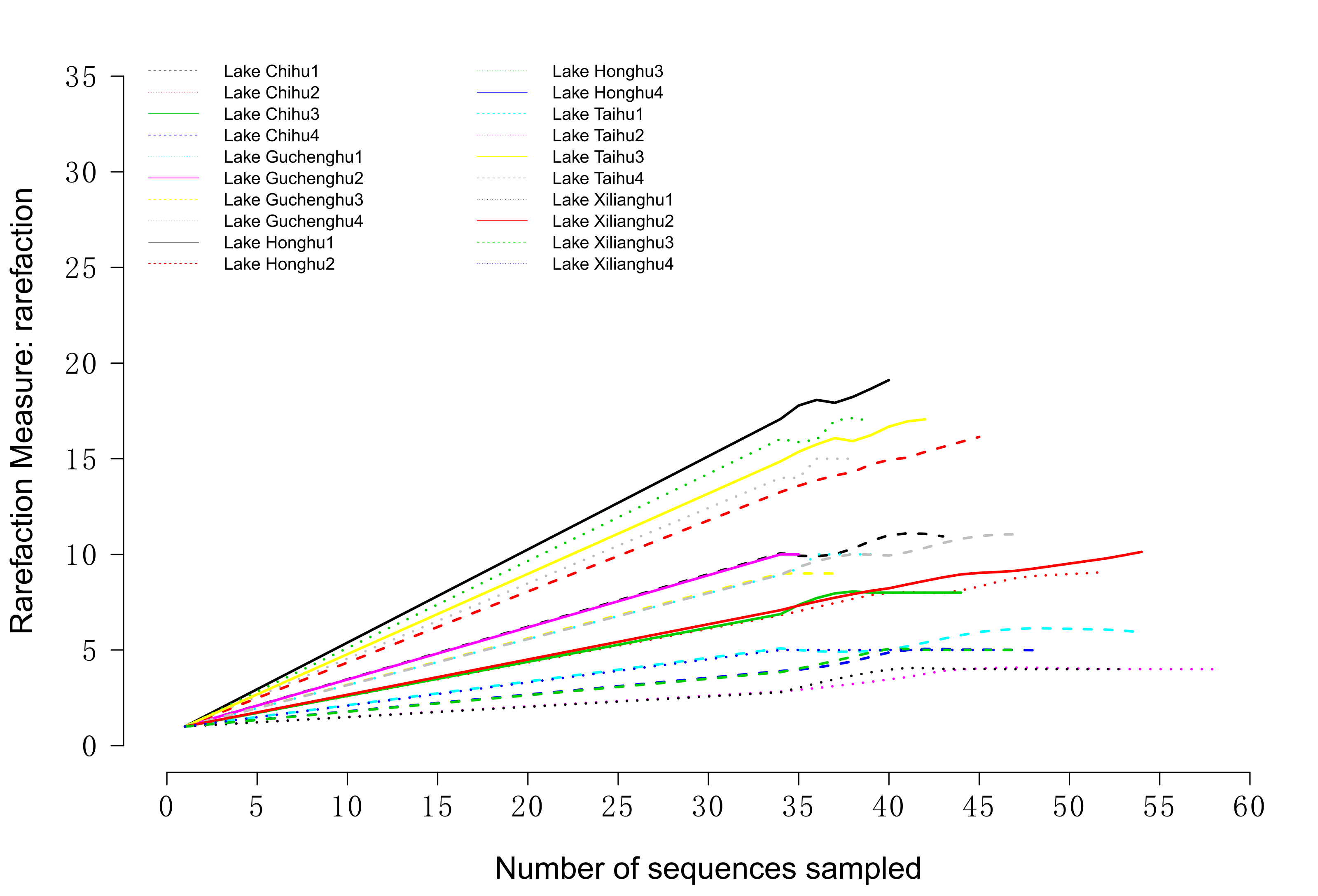

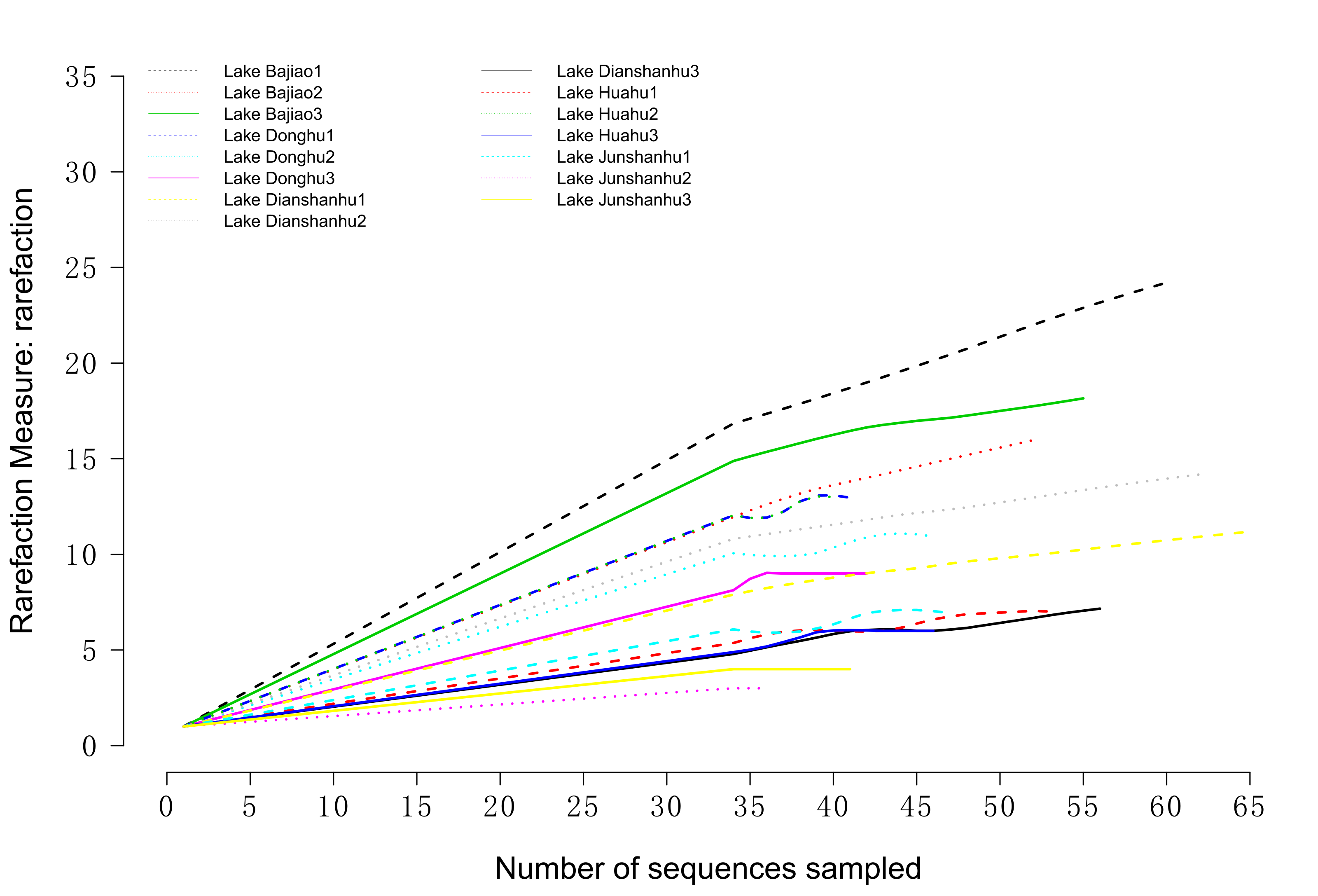


**Figure S2**

**Table S1** Primers and conditions for qualitative and quantitative PCR.

|  | Target gene | Primer set | Primer sequence (5'–3') | Size | Thermal profile | Reference |
| --- | --- | --- | --- | --- | --- | --- |
| PCR | Archaeal *amoA* | Arch-amoAF | STAATGGTCTGGCTTAGACG | 635 bp | 95°C for 5 min; 35 cycles consisting of 94°C for 30 s, 53°C for 45 s, and 72°C for 60 s; and 72°C for 10 min | Francis *et al*. (2005) |
|  |  | Arch-amoAR | GCGGCCATCCATCTGTATGT |  |  |  |
|  | Bacterial *amoA* | amoA-1F | GGGGTTTCTACTGGTGGT | 491 bp | 95°C for 5 min; 35 cycles consisting of 94°C for 30 s, 55°C for 40 s, and 72°C for 45 s; and 72°C for 10 min | Rotthauwe *et al*. (1997) |
|  |  | amoA-2R | CCCCTCKGSAAAGCCTTCTTC |  |  |  |
| Real-time PCR | Archaeal *amoA* | Arch-amoAF | STAATGGTCTGGCTTAGACG | 635 bp | 95°C for 3 min; 40 cycles of 95°C for 15 s, 53°C for 20 s, and 72°C for 30 s; followed by a plate read at 87°C | This study |
|  |  | Arch-amoAR | GCGGCCATCCATCTGTATGT |  |  |  |
|  | Bacterial *amoA* | amoA-1F | GGGGTTTCTACTGGTGGT | 491 bp | 95°C for 3 min; 40 cycles of 95°C for 15 s, 55°C for 20 s, and 72°C for 30 s; followed by a plate read at 89°C | This study |
|  |  | amoA-2R | CCCCTCKGSAAAGCCTTCTTC |  |  |  |

**Table S2** Abundance and diversity indices of clone libraries of archaeal *amoA* genes in different sampling sites

| Sampling site | Number of clones | OTUs | Chao1 | Shannon | Simpson | Abundance  (10^4^ copies g^-1^) |
| --- | --- | --- | --- | --- | --- | --- |
| BJH1 | 43 | 7 | 8.5 | 1.34 | 0.33 | 333.34 |
| BJH2 | 57 | 5 | 5 | 1.18 | 0.38 | 75.13 |
| BJH3 | 31 | 6 | 6 | 1.26 | 0.37 | 13.87 |
| CH1 | 46 | 6 | 7 | 1.29 | 0.33 | 3.90 |
| CH2 | 45 | 18 | 24 | 2.48 | 0.11 | 2.28 |
| CH3 | 47 | 3 | 3 | 1.06 | 0.34 | 1.07 |
| CH4 | ND | ND | ND | ND | ND | 0.73 |
| DH1 | 46 | 6 | 9 | 0.59 | 0.75 | 2.39 |
| DH2 | 47 | 5 | 5 | 1.29 | 0.32 | 2.26 |
| DH3 | 46 | 5 | 6 | 0.96 | 0.49 | 1.85 |
| DSH1 | 34 | 5 | 6 | 1.19 | 0.34 | 0.64 |
| DSH2 | 47 | 9 | 14 | 1.37 | 0.37 | 0.87 |
| DSH3 | 47 | 5 | 5.5 | 0.85 | 0.55 | 0.45 |
| GCH1 | 39 | 2 | 2 | 0.20 | 0.90 | 0.02 |
| GCH2 | ND | ND | ND | ND | ND | 1.36 |
| GCH3 | 33 | 11 | 13.5 | 2.02 | 0.15 | 0.75 |
| GCH4 | 50 | 18 | 36 | 2.58 | 0.08 | 0.96 |
| HOH1 | ND | ND | ND | ND | ND | 0.79 |
| HOH2 | 48 | 3 | 4 | 0.20 | 0.92 | 0.64 |
| HOH3 | 48 | 3 | 3 | 0.33 | 0.84 | 1.93 |
| HOH4 | ND | ND | ND | ND | ND | 0.87 |
| HUH1 | 45 | 4 | 4 | 0.78 | 0.59 | 1.16 |
| HUH2 | 47 | 4 | 4.5 | 0.38 | 0.84 | 1.41 |
| HUH3 | 36 | 1 | 1 | 0.00 | 1.00 | 1.41 |
| JSH1 | 49 | 10 | 12 | 1.80 | 0.20 | 1.44 |
| JSH2 | 38 | 7 | 13 | 1.43 | 0.27 | 0.54 |
| JSH3 | 44 | 11 | 32 | 1.73 | 0.24 | 1.14 |
| TH1 | 48 | 11 | 13 | 1.77 | 0.26 | 5.30 |
| TH2 | 46 | 3 | 3 | 0.95 | 0.42 | 0.59 |
| TH3 | 66 | 11 | 14 | 0.77 | 0.60 | 4.05 |
| TH4 | 53 | 6 | 12 | 1.88 | 0.20 | 0.68 |
| XLH1 | 47 | 7 | 10 | 1.31 | 0.37 | 1.90 |
| XLH2 | 49 | 7 | 10 | 1.33 | 0.35 | 2.03 |
| XLH3 | 39 | 5 | 5 | 1.16 | 0.38 | 0.77 |
| XLH4 | 43 | 7 | 8 | 1.63 | 0.22 | 1.38 |

BJH: Lake Bajiaohu; CH: Lake Chihu; DH: Lake Donghu; DSH: Lake Dianshanhu; GCH: Lake Guchenghu; HOH: Lake Honghu; HUH: Lake Huahu; JSH: Lake Junshanhu; TH: Lake Taihu; XLH: Lake Xilianghu

ND: no data

**Table S3** Abundance and diversity indices of clone libraries of bacterial *amoA* genes in different sampling sites

| Sampling site | Number of clones | OTUs | Chao1 | Shannon | Simpson | Abundance  (10^4^ copies g^-1^) |
| --- | --- | --- | --- | --- | --- | --- |
| BJH1 | 60 | 24 | 45.0 | 2.66 | 0.10 | 10.37 |
| BJH2 | 52 | 16 | 43.5 | 2.19 | 0.14 | 4.77 |
| BJH3 | 55 | 18 | 19.3 | 2.63 | 0.07 | 2.75 |
| CH1 | 43 | 11 | 26.0 | 1.67 | 0.30 | 1.11 |
| CH2 | 52 | 9 | 12.3 | 1.00 | 0.59 | 0.55 |
| CH3 | 44 | 8 | 11.0 | 1.67 | 0.21 | 0.95 |
| CH4 | 48 | 5 | 8.0 | 0.58 | 0.73 | 0.49 |
| DH1 | 41 | 13 | 20.0 | 2.13 | 0.14 | 5.36 |
| DH2 | 46 | 11 | 16.0 | 1.90 | 0.19 | 2.40 |
| DH3 | 42 | 9 | 9.8 | 1.62 | 0.26 | 1.32 |
| DSH1 | 65 | 11 | 14.3 | 1.38 | 0.43 | 0.32 |
| DSH2 | 62 | 14 | 19.0 | 1.98 | 0.21 | 0.78 |
| DSH3 | 56 | 7 | 17.0 | 0.74 | 0.68 | 0.22 |
| GCH1 | 39 | 10 | 31.0 | 1.37 | 0.42 | 0.35 |
| GCH2 | 35 | 10 | 31.0 | 1.64 | 0.27 | 0.81 |
| GCH3 | 37 | 9 | 14.0 | 1.33 | 0.42 | 0.43 |
| GCH4 | 38 | 15 | 30.0 | 2.02 | 0.23 | 0.96 |
| HOH1 | 40 | 19 | 41.0 | 2.66 | 0.07 | 1.32 |
| HOH2 | 45 | 16 | 43.5 | 2.17 | 0.16 | 1.22 |
| HOH3 | 39 | 17 | 22.1 | 2.39 | 0.13 | 3.36 |
| HOH4 | 34 | 9 | 30.0 | 1.36 | 0.38 | 0.90 |
| HUH1 | 53 | 7 | 10.0 | 0.90 | 0.58 | 0.41 |
| HUH2 | 40 | 13 | 58.0 | 1.81 | 0.25 | 0.60 |
| HUH3 | 46 | 6 | 7.5 | 0.93 | 0.54 | 0.47 |
| JSH1 | 47 | 7 | 10.0 | 0.81 | 0.65 | 0.56 |
| JSH2 | 36 | 3 | 3.0 | 0.41 | 0.79 | 0.26 |
| JSH3 | 41 | 4 | 4.0 | 0.56 | 0.73 | 0.35 |
| TH1 | 54 | 6 | 9.0 | 0.96 | 0.52 | 0.30 |
| TH2 | 58 | 4 | 5.0 | 0.68 | 0.61 | 0.22 |
| TH3 | 42 | 17 | 29.0 | 2.51 | 0.09 | 1.37 |
| TH4 | 47 | 11 | 16.0 | 1.39 | 0.44 | 0.17 |
| XLH1 | 53 | 4 | 5.0 | 0.71 | 0.59 | 0.90 |
| XLH2 | 54 | 10 | 20.5 | 1.12 | 0.53 | 0.47 |
| XLH3 | 47 | 5 | 8.0 | 0.54 | 0.76 | 0.31 |
| XLH4 | 46 | 5 | 5.5 | 0.67 | 0.69 | 0.59 |

BJH: Lake Bajiaohu; CH: Lake Chihu; DH: Lake Donghu; DSH: Lake Dianshanhu; GCH: Lake Guchenghu; HOH: Lake Honghu; HUH: Lake Huahu; JSH: Lake Junshanhu; TH: Lake Taihu; XLH: Lake Xilianghu

ND: no data
